# Sustained capillary enlargement induced by angiogenic gene therapy does not support post-ischemic muscle recovery of hyperlipidemic mice

**DOI:** 10.3389/fbioe.2025.1512962

**Published:** 2025-05-27

**Authors:** Galina Wirth, Greta Juusola, Hanne Laakso, Nihay Laham-Karam, Seppo Ylä-Herttuala, Petra Korpisalo

**Affiliations:** ^1^ Heart Center, Kuopio University Hospital, Kuopio, Finland; ^2^ A. I. Virtanen Institute for Molecular Sciences, University of Eastern Finland, Kuopio, Finland

**Keywords:** hyperlipidemia, ischemia, adenovirus, VEGF, gene therapy, capillary enlargement, muscle damage, edema

## Abstract

**Background:**

Hyperlipidemia is known to impair endothelial function. We have recently shown that hyperlipidemia also blunts native post-ischemic capillary enlargement that is important for efficient skeletal muscle recovery from ischemia as it supports the recovery of arterial driving pressure and through intussusception increases capillary density. The correction of capillary reactivity under hyperlipidemia could, therefore, improve post-ischemic skeletal muscle recovery. This study tested the ability of adenoviral (Ad) vascular endothelial growth factor (VEGF) gene therapy to rescue capillary enlargement and improve post-ischemic muscle repair in hyperlipidemic mice.

**Methods:**

AdVEGF or AdLacZ-control vector were delivered into the calf muscles of aged, hyperlipidemic LDLR^−/−^ApoB^100/100^ mice (n = 58) after induction of acute ischemia. The effects of AdVEGF on capillary phenotype, tissue edema, restoration of blood flow parameters, microvascular hemoglobin oxygenation and tissue damage/regeneration were evaluated using immunohistological analyses, magnetic resonance imaging, contrast-enhanced ultrasound imaging, photoacoustic imaging and histological analyses, respectively, up to 29 days after induced ischemia and gene transfer.

**Results:**

It was found that AdVEGF gene therapy was able to promote capillary enlargement (P < 0.05) that led to recovery of arterial driving pressure in ischemic LDLR^−/−^ApoB^100/100^ muscles. However, capillary enlargement induced by AdVEGF in the hyperlipidemic mice was delayed, had a long-lasting effect (P < 0.05) and did not promote intussusception. Instead, side-effects of VEGF-induced capillary enlargement, i.e., tissue edema (P < 0.01) and subsequently delayed blood flow recovery (P < 0.05), aggravated ischemic tissue damage (P < 0.01).

**Conclusion:**

Hyperlipidemia or old age did not seem to impair AdVEGF-induced capillary enlargement. However, regarding the side-effects of capillary enlargement, therapies trying to promote post-ischemic skeletal muscle recovery through angiogenesis should consider not only capillary size or density but also timing and dynamics of the capillary changes.

## 1 Introduction

Angiogenic gene therapy has been proposed as the means to improve capillary reactivity in response to ischemia and to promote capillary enlargement in vascularized tissue (Rissanen et al., 2005; Korpisalo et al., 2011). Although the therapeutic efficacy of many angiogenic factors, such as members of the vascular endothelial growth factor (VEGF) family, has been extensively tested in peripheral ischemia models (Rissanen et al., 2005; Gowdak et al., 2000; Korpisalo et al., 2008a; Korpisalo et al., 2008b; Olea et al., 2009), clinical trials of angiogenic therapy have so far produced few positive results (Ylä-Herttuala et al., 2017; Annex and Cooke, 2021; Peeters et al., 2024). The preclinical testing of pro-angiogenic strategies has typically been done in young and healthy animals. However, patients with advanced age and underlying co-morbidities could respond differently to therapeutic stimuli than young and healthy animals (Dragneva et al., 2013). Therefore, the use of clinically relevant disease models is imperative and may provide better understanding of the negative results of clinical studies (Annex and Cooke, 2021).

Hyperlipidemia is known to impair endothelial function in both peripheral and coronary circulation (Davignon and Ganz, 2004; Granger et al., 2010; Benincasa et al., 2022; Kavurma et al., 2022; Higashi, 2023) and could, therefore, interfere with angiogenic gene therapy. Endothelial dysfunction associated with hyperlipidemia has been attributed to reduced vasodilation response to various stimuli. Indeed, in hyperlipidemia, increased shear stress or hypoxia driven signaling results in lower vasodilation, due to decreased bioavailability of nitric oxide (Davignon and Ganz, 2004; Granger et al., 2010; Benincasa et al., 2022). Also, hyperlipidemia has been associated with an increase in ROS production, thereby negatively affecting the endothelium by increasing endothelial cell oxidative stress (Davignon and Ganz, 2004; Higashi, 2023). We have recently shown that hyperlipidemia also blunts native post-ischemic capillary enlargement that was identified to be important for efficient skeletal muscle recovery from ischemia (Wirth et al., 2023). Early post-ischemic capillary enlargement, through increasing blood flow in newly opened collateral arteries, could support collateral maturation and the recovery of arterial driving pressure (Wirth et al., 2023). After post-ischemic recovery of arterial driving pressure, intussusceptive splitting of enlarged capillaries promotes tissue regeneration through increasing capillary density and improving tissue oxygenation (Wirth et al., 2023). We have also shown that the impairment of native capillary dynamics under hyperlipidemia results in the inability to recover arterial driving pressure, delays in both tissue oxygenation recovery and muscle regeneration as well as in the formation of chronic atrophic damage (Wirth et al., 2023). Based on these observations, correction of impaired capillary reactivity under hyperlipidemia could, therefore, act to improve post-ischemic skeletal muscle recovery.

This study tested the ability of adenoviral (Ad) VEGF gene therapy to rescue capillary enlargement and improve post-ischemic muscle repair in aged, hyperlipidemic LDLR^−/−^ApoB^100/100^ mice. The effects of VEGF gene therapy on capillary phenotype, tissue edema, restoration of blood flow parameters, microvascular hemoglobin oxygenation and tissue damage/regeneration were studied for up to 29 days after interventions using immunohistology, magnetic resonance imaging, contrast-enhanced ultrasound imaging, photoacoustic imaging and histological analyses, respectively.

## 2 Materials and methods

### 2.1 Animal model and gene transfer

All animal experiments were approved by The Finnish National Animal Experiment Board (ELLA) (license number: ESAVI/5343/04.10.07/2014) and followed the guidelines from Directive 2010/63/EU of the European Parliament on the protection of animals used for scientific purposes or the NIH Guide for the Care and Use of Laboratory Animals. The animals in the present study were done at the same time using the same methods as a set of aged, healthy mice and aged, hyperlipidemic mice without gene transfer which were previously published (Wirth et al., 2023). Hyperlipidemic mice deficient of LDL receptor and expressing only apolipoprotein B100 (LDLR^−/−^ApoB^100/100^ female mice, C57Bl/6J background bred in-house, n = 58), with an average age of 9 months, were used in this study. In addition to having a human-like lipoprotein profile, these animals normally have a 3-fold increase in blood cholesterol levels, predisposing them to develop atherosclerosis even on a regular chow diet (Véniant et al., 1998; Heinonen et al., 2007). Mice underwent permanent ligation of both the common femoral artery and vein proximal to the origin of the profound femoral artery of the right hindlimb, resulting in acute distal ischemia. Post-operatively, mice were randomized and allocated into two groups for adenoviral vector transfers. Adenoviral vectors, produced in the National Virus Vector Laboratories at the University of Eastern Finland, either expressing human VEGF-A_165_ (AdVEGF) or beta-galactosidase (AdLacZ) control gene (Korpisalo et al., 2011) were delivered as a single injection into the ischemic posterior calf muscles. A total dose of 2 x 10^12^ vp/mL in 50 µL 0.9% NaCl was evenly distributed along the injection route, starting from the proximal end towards the distal end of the calf muscle, using 0.33 mL insulin syringe. Mice were anesthetized with isoflurane inhalation (induction: 4.5% isoflurane, 450 mL/min air, maintenance: 2.5% isoflurane, 250 mL/min air; Baxter International, Deerfield, IL, United States) during surgery and ultrasound imaging. After surgery, mice received subcutaneously analgesia (Rimadyl, 10 mg/kg, Pfizer, Dublin, Ireland). The animals were fed on a regular chow diet and kept in standard housing conditions in the National Animal Laboratory Center of the University of Eastern Finland, Kuopio, Finland.

### 2.2 Magnetic resonance imaging of edema

Tissue edema was assessed with MRI using 7-T Bruker PharmaScan Magnet (Bruker BioSpin, Ettlingen, Germany) interfaced to Paravision 5.1, using a quadrature volume coil as a transmitter and a 20 mm single loop surface coil as a receiver pre-operatively and at 1, 4, 7 and 29 days after femoral artery ligation and gene transfer. Mice were anesthetized with isoflurane (Baxter Oy, Helsinki, Finland) in 70% N_2_: 30% O_2_ for imaging and placed on a holder carefully stretching the hindlimbs straight over the imaging coil. A pneumatic pillow was placed under the mouse to monitor respiration using a small animal monitoring unit (Model 1,025, SA Instruments, Stony Brook, NY, United States). A warm water circulating pad was set over the mouse to maintain a temperature close to the physiological level. The legs were imaged using multi-slice T2-weighted fast spin echo images [repetition time (TR) = 2 s, effective echo time (TE) = 22 m, echo train length (ETL) = 4, field of view (FOV) = 20 × 20 mm^2^, matrix = 256 × 256, two averages, 20 slices, slice thickness 1 mm]. From these images, the whole leg cross section area (mm^2^), used to measure edema build-up, was determined by hand-drawn regions of interest (ROIs) at a medial position of the calf muscle and expressed as an average of ROIs from three consecutive slices, using Aedes software package (aedes.uef.fi) on Matlab platform (Math Works, Natick, MA).

### 2.3 Ultrasound imaging of skeletal muscle perfusion

Resting blood flow in calf muscles was monitored and quantitatively measured with Acuson Sequoia 512 system and a 15L8 transducer (Siemens, Malvern, PA, United States). Contrast enhanced Power Doppler (CED) and Cadence contrast pulse sequencing (CEU) applications were used to differentiate between blood flow in large arterial/venous vessels and the microvasculature, as previously described (Wirth et al., 2023). Measurements were made pre-operatively and at 0, 4, 7 and 29 days after surgery and gene transfer. Briefly, transverse plane perfusion video clips (20 s in length, 15 clip frames/1 s) were captured immediately following injection of 50 μL bolus of a second-generation contrast agent (a sulfur hexafluoride gaseous core in a phospholipid shell, approx. 2 x 10^8^ bubbles/mL, mean size 2.5 µm, SonoVue, Bracco, Milan, Italy) via the jugular vein. Arterial/venous and microvascular skeletal muscle blood flow were quantified with Datapro software (v2.13, Noesis, Courtaboeuf, France) using CED and CEU peak signal intensities (dB; relative to both blood flow and volume) of the video clips, respectively. The level of arterial driving pressure was determined based on CEU-contrast arrival time measurements, i.e., the time (in seconds) from bolus injection to the arrival of the contrast agent into the imaging plane (Wirth et al., 2023; Rissanen et al., 2008). Maximal arrival time was recorded in instances in which the contrast agent did not arrive to the imaging plane within the 20-seconds-time measurement window. Ultrasound data analysis was performed in a blind manner.

### 2.4 Photoacoustic imaging of microvascular oxygen saturation of hemoglobin

Microvascular oxygen saturation of hemoglobin in calf muscles was monitored and quantitatively measured with Vevo 2100 LAZR system and a linear-array transducer LZ250 (VisualSonics Inc., Toronto, ON, Canada) in a photoacoustic (PA) mode (frequency = 21 MHz, PA gain = 50 dB, 2D gain = 18 dB, depth = 20 mm, width = 23.04 mm and dual laser wavelength = 750/850 nm) as previously described (Wirth et al., 2023). Measurements were performed pre-operatively and at 0, 4, 7 and 29 days after surgery and gene transfer. Muscle level average microvascular oxygen saturation of hemoglobin (mHbO_2_%) was quantified with Vevo LAB software (v1.7.2, VisualSonics Inc., Toronto, ON, Canada) from at least 30 transverse plane PA clip frames.

### 2.5 Histology and immunohistochemistry

Mice were euthanized with carbon dioxide and perfusion-fixed with 1% paraformaldehyde (PFA) in phosphate-buffered saline (PBS) through the left ventricle. After dissection, posterior calf muscles were immersion-fixed in 4% PFA in 7.5% sucrose (pH 7.4) for 4 h and kept in 15% sucrose in H_2_O (pH 7.4) overnight. After fixation, calf muscle samples were embedded in paraffin and transversely cut to 4 µm thick sections used for immunohistochemical and hematoxylin-eosin (HE) staining.

To evaluate post-ischemic muscle capillary reactivity, endothelium was immunostained with a rat monoclonal primary antibody against CD31 [platelet endothelial cell adhesion molecule (PECAM-1), dilution 1:25, MEC 13.3, BD Biosciences Pharmingen, San Diego, CA, United States] and biotinylated rabbit anti-rat secondary antibody (dilution 1:200, Vector Laboratories, Burlingame, CA, United States). To visualize the immunoreactivity, avidin-biotin-horseradish peroxidase system (Vector Laboratories, Burlingame, CA, United States) and 3,3′-Diaminobenzidine (DAB) color substrate (Zymed, San Francisco, CA, United States) were used. Tyramide signal amplification system (TSA, dilution 1:50, Biotin System, PerkinElmer, Shelton, CT, United States) was used to enhance signal intensity. Images were taken with Nikon H550L light microscope (Tokyo, Japan) and NIS-Elements advanced research imaging software at ×20 magnification. The following parameters were measured from the immunostained images: capillary area (endomysium localized CD31^+^ mean vessel luminal area in µm^2^), capillary density (endomysium localized CD31^+^ vessels/muscle area mm^2^), capillary size distribution [endomysium localized CD31^+^ vessels with a luminal area more than 33 μm^2^ reported as a percentage of the total vessel amount/time point as previously described (Wirth et al., 2023)] and total capillary area (capillary density/capillary area in mm^2^). These measurements were calculated from two to three fields using NIS-Elements software (v4.51.01, Nikon, Tokyo, Japan). Only areas with myofiber histopathological changes were selected for the evaluation of CD31-based capillary reactivity as previously described (Lee et al., 2020). CD31-stained calf muscle sections deriving from intact hindlimbs (n = 8) were used to determine capillary parameters at baseline.

To assess post-ischemic myofiber responses, calf muscle sections were stained with HE as previously described (Wirth et al., 2023). The sections were then first carefully examined with Olympus AX-70 light microscope (Olympus Optical, Tokyo, Japan) at ×20 magnification, after which analysis images were obtained at ×1.25 magnification using analySIS imaging software (v3.00, Soft Imaging System GmbH, Muenster, Germany). Six irregularly distributed areas representing 1) normal area, 2) rounded myofibers, 3) necrosis, 4) early regenerative changes, 5) advanced regeneration and 6) late regeneration were measured as previously characterized in detail (Wirth et al., 2023). The damaged muscle area was calculated as the sum of areas 2–3. The regenerative muscle area was calculated as the sum of areas 4–6. In addition, area with mixed myofiber atrophy was identified by the presence of very small angulated myofibers often devoid of nuclei in addition to scattered very small rounded myofibers with/without peripherally oriented condensed, dark nuclei. Fibrotic area was defined by the presence of connective tissue together with scattered severely atrophied myofibers, of which some were almost reduced to clusters of nuclei, occasional fibroblasts, and mononuclear cells. Mononuclear pockets were considered large clusters of dark basophilic mononuclear cells located within the skeletal muscle tissue. All defined areas were reported as a percentage of the whole cross-sectional muscle area. All measurements were performed in a blind manner.

### 2.6 Statistical analysis

Statistical analyses were performed using SPSS software (v27.0 for Windows, SPSS Inc., Chicago, IL, United States). Results were expressed as mean ± standard error of the mean (SEM). The Kruskal–Wallis test followed by the Mann-Whitney U test were applied for the assessment of significant changes between the control and treated group as well as within each group. The difference in the prevalence of macroscopical ischemic symptoms between the two groups was assessed using the Chi-squared test. Differences were considered significant at ^*^, ^#^P < 0.05, ^**^, ^##^P < 0.01, ^***^, ^###^P < 0.001.

## 3 Results

### 3.1 Post-ischemic AdVEGF gene therapy induces long-lasting capillary enlargement in elderly, hyperlipidemic LDLR^−/−^ApoB^100/100^ mice

**FIGURE 1:**
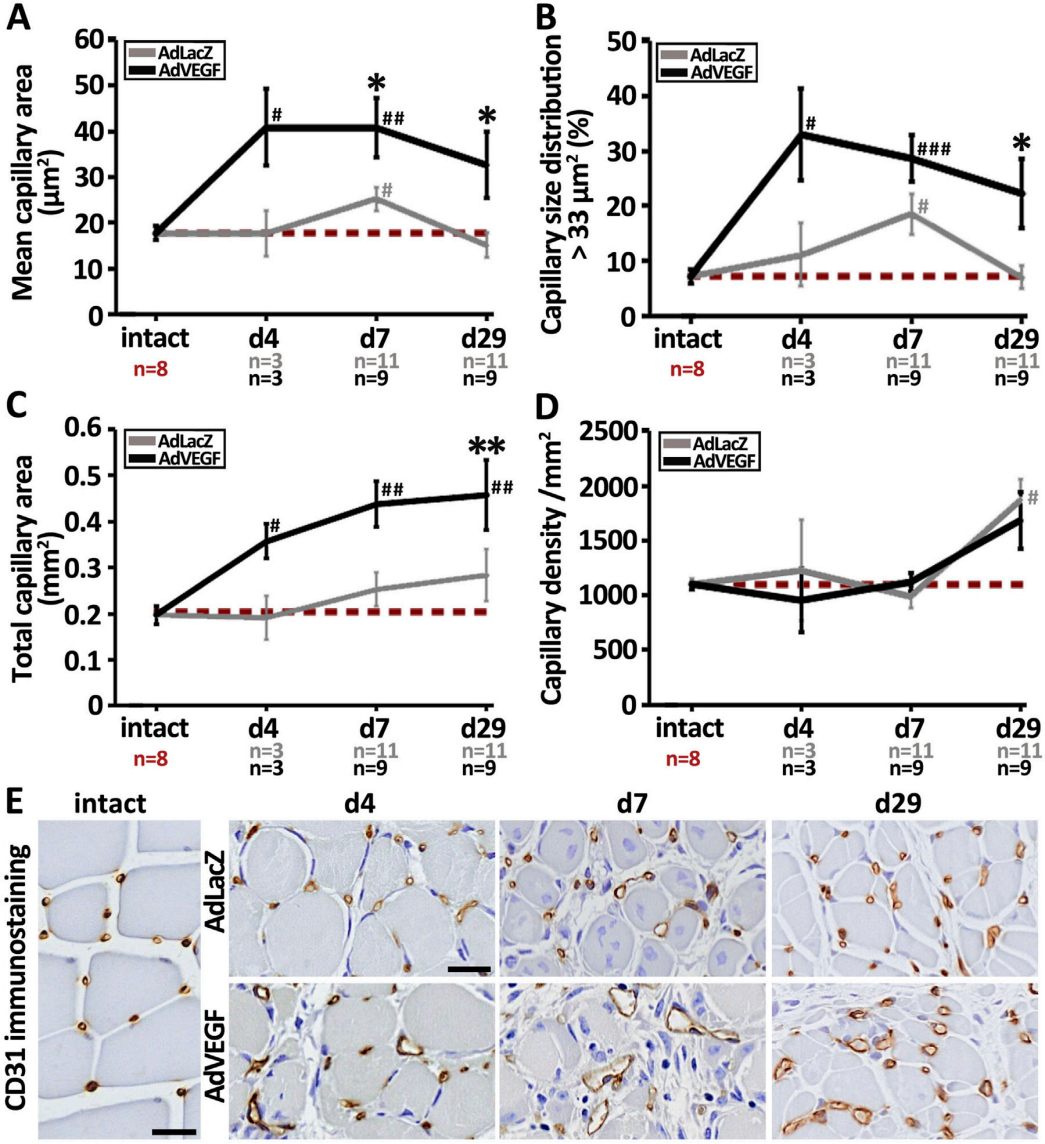
AdVEGF gene transfer significantly increases capillary area in elderly, hyperlipidemic LDLR^−/−^ApoB^100/100^ mice. CD31-based quantification of **(A)** capillary area, **(B)** capillary size distribution and **(C)** total capillary area demonstrated that the capillaries in the AdVEGF-transduced ischemic calf muscles underwent long-lasting enlargement affecting on average about 30% of the capillaries, which significantly increased the total capillary area on day 29 as compared to control values. However, no increase in **(D)** capillary density in the AdVEGF group was observed when compared to the control. **(E)** Representative CD31 (brown) immunohistochemical stainings displaying the morphological appearance of capillaries in intact skeletal muscle and in AdLacZ- and AdVEGF-transduced ischemic calf muscle 4, 7 and 29 days after gene transfer. Many of the enlarged vessels in the AdVEGF group had aberrant wall structure as compared to the conventional round shape of capillaries in intact and control muscles. Scale bars: 20 µm. Dashed, red line in **(A–D)** indicates the baseline level. Statistical significance is indicated by ^*^, ^#^p < 0.05, ^**^, ^##^p < 0.01, ^###^p < 0.001. Comparison to AdLacZ is indicated with ^*^ and comparison to intact muscle with ^#^.

As quantified from CD31-immunostainings of ischemic muscle samples, AdVEGF gene transfer significantly increased mean capillary area of hyperlipidemic LDLR^−/−^ApoB^100/100^ mice as compared to the AdLacZ control (Figure 1A). A statistically significant increase was observed 7 days after the gene transfer although there was a visible enlargement present already on day 4 in some capillaries. In contrast to previous reports (Rissanen et al., 2005; Korpisalo et al., 2011; Kholová et al., 2007), the enlargement in AdVEGF stimulated capillaries persisted at least until the end of the follow-up on day 29. At that time, close to 30% of the capillaries in the AdVEGF group were still enlarged (Figure 1B). Total capillary area in the AdVEGF group was significantly increased only on day 29 as compared to AdLacZ control (Figure 1C). Capillary density was not affected by the AdVEGF gene transfer as it remained unchanged throughout the 29-day follow-up when compared to control values (Figure 1D). On days 4–7, many of the enlarged vessels in the AdVEGF group seemed aberrant in structure (Figure 1E). In contrast, on day 29 the enlarged vessels in AdVEGF muscles were structurally round and displayed thick walls. The angiogenic effect of the AdVEGF gene transfer in the ischemic hyperlipidemic muscle, hence, mainly seemed to result in long-term enlargement of pre-existing capillaries without increasing capillary number.

### 3.2 Post-ischemic capillary enlargement induced by AdVEGF gene therapy is related to severe tissue edema

**FIGURE 2:**
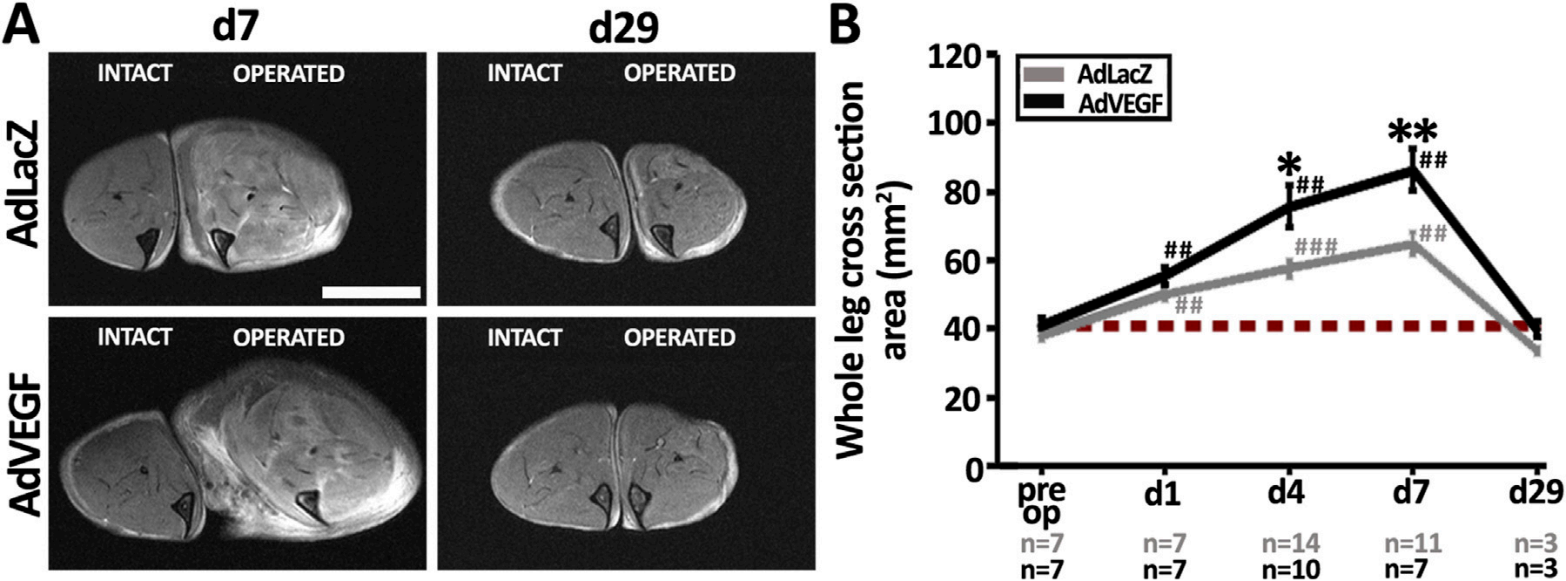
Severe edema is a side-effect of AdVEGF-induced capillary enlargement. **(A)** MRI-based T2-weighted images displaying the formation of mild, intramuscular edema vs. severe, widespread edema in the AdLacZ-vs. AdVEGF-transduced muscle on day 7, respectively, and the disappearance of edema in both groups by day 29 post-surgery and gene transfer. **(B)** MRI-based quantification of the whole leg cross sectional area (mm^2^) confirmed significant swelling of the ischemic legs starting already on day 1. AdVEGF gene transfer worsened tissue edema during days 4–7. Scale bar: 5 mm. Dashed, red line in **(B)** indicates the baseline level. Statistical significance is indicated by ^*^p < 0.05, ^**^, ^##^p < 0.01, ^###^p < 0.001. Comparison to AdLacZ is indicated with ^*^ and comparison to pre op values with ^#^.

T2-weighted MRI was used to visualize edema build-up after the gene transfers (Figure 2A). On day 7, the operated hindlimb in the AdLacZ group showed mostly intramuscular edema formation as compared to the intact hindlimb. At the same time, the operated hindlimb in the AdVEGF group displayed not only intramuscular edema but also massive subcutaneous fluid deposits. When quantified as total hindlimb area, the MR-images in both groups showed significant swelling as compared to their respective contralateral intact legs (Figure 2B). However, significantly more hindlimb swelling was observed in the AdVEGF limbs as compared to the AdLacZ control limbs. By day 29, there were no signs of edema in either group. In the ischemic limbs of the hyperlipidemic mice AdVEGF gene transfer thus caused transient edema formation that on average doubled the cross-sectional hindlimb area on days 4–7.

### 3.3 Recovery of skeletal muscle microvascular blood flow after ischemia is delayed in the elderly, hyperlipidemic LDLR^−/−^ApoB^100/100^ mice with AdVEGF gene transfer

**FIGURE 3:**
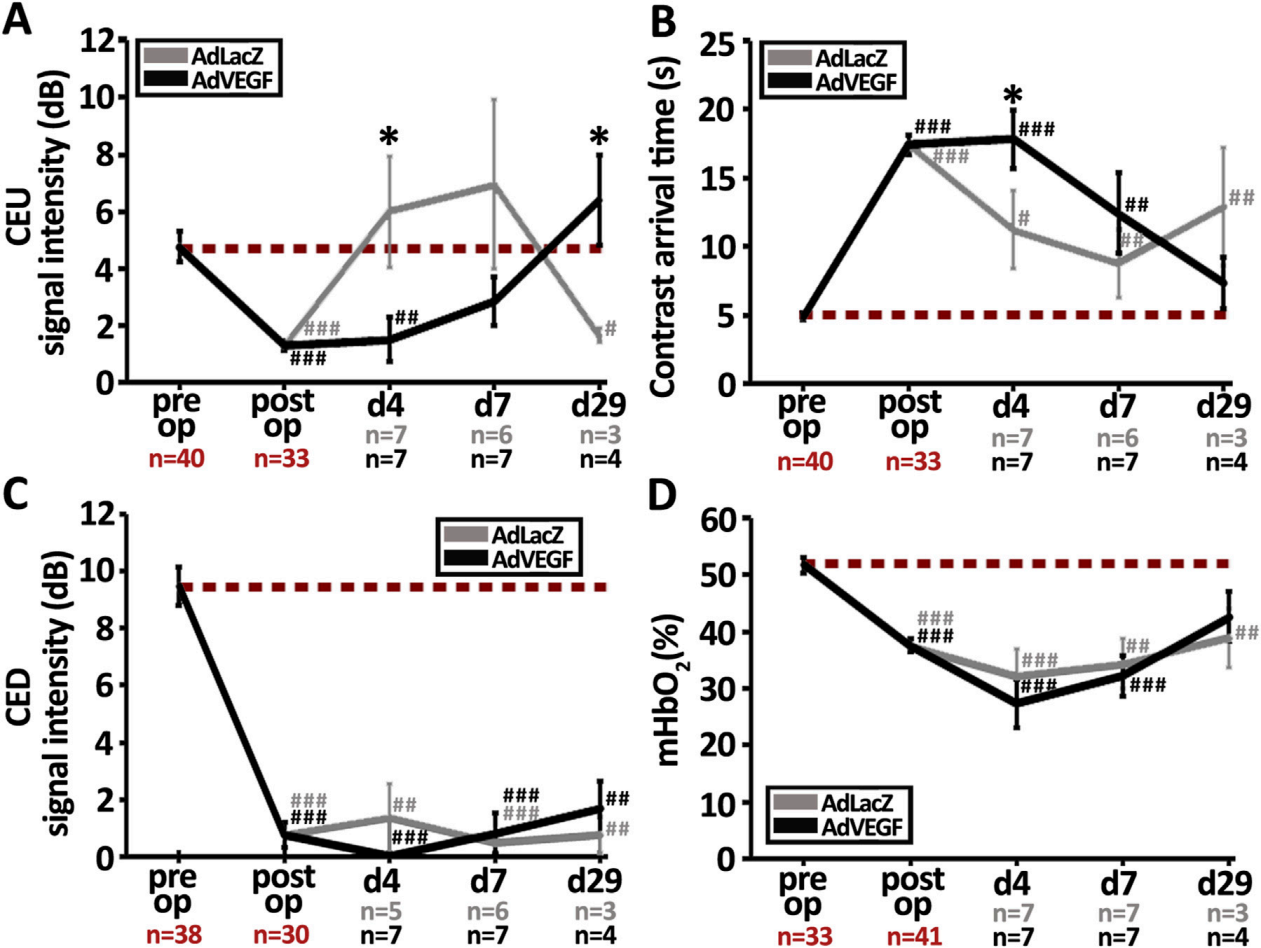
AdVEGF gene transfer delays microvascular blood flow recovery but manages to recover arterial driving pressure. Quantification of changes in **(A)** microvascular blood flow and **(B)** contrast arrival time from CEU. The AdLacZ control showed recovery of tissue level microvascular blood flow on day 4 but no recovery of arterial driving pressure, as indicated by significantly increased contrast arrival time throughout the 29-day follow-up. In the AdVEGF group, microvascular blood flow recovery was delayed until day 7 but arterial driving pressure recovered by day 29. Quantification of **(C)** macrovascular blood flow from CED displayed no recovery in either group by d29. **(D)** PAI-based quantification of microvascular hemoglobin oxygenation (mHbO_2_%) showed recovery only in the AdVEGF group on d29. Dashed, red line in **(A–D)** indicates the baseline level. Statistical significance is indicated by ^*^, ^#^p < 0.05, ^##^p < 0.01, ^###^p < 0.001. Comparison to AdLacZ is indicated with ^*^ and comparison to pre op values with ^#^.

Unlike previously in young and healthy animals, gene transfer of AdVEGF in aged and diseased mice did not improve muscle blood flow in this study. High-resolution CEU and CED ultrasound imaging were performed to differentiate between blood flow in the microvasculature (CEU) and large arterial/venous vessels (CED). As expected, femoral artery ligation significantly reduced microvascular flow similarly in both groups (Figure 3A, P < 0.001). 4 days after the operation, CEU contrast signal intensity was restored to baseline level in the AdLacZ group, but not in the AdVEGF group, indicating that AdVEGF transduced muscles had a slower microvascular flow recovery than the control muscles (Figure 3A, P < 0.05). The average CEU signal intensity in the AdVEGF group reached baseline values only by day 29. Arrival time of CEU contrast agent inversely reflects the level of arterial driving pressure in the operated hindlimbs. Femoral artery ligation significantly increased CEU arrival time in both groups (Figure 3B, P < 0.05). In the AdLacZ control, arrival time remained significantly increased throughout the follow-up, indicating that arterial driving pressure did not recover in the AdLacZ control. In contrast, CEU arrival time in the AdVEGF group gradually decreased, recovering to baseline levels by day 29. Large arterial/venous CED flow significantly decreased upon the ischemic insult and remained significantly reduced equally in both groups during the 29-day follow-up (Figure 3C, P < 0.01). PAI-based quantification of mHbO_2_% displayed that femoral artery ligation significantly reduced ischemic muscle microvascular hemoglobin oxygenation (Figure 3D, P < 0.001). During the later follow-up, PAI-based quantification of mHbO_2_% showed no significant differences between the treatment groups and almost no recovery to baseline levels during the follow-up, except with AdVEGF on day 29. Based on the blood flow parameters, AdVEGF gene transfer therefore delayed post-ischemic recovery of microvascular blood flow. Nevertheless, on day 29 the AdVEGF group displayed significantly higher microvascular blood flow than the AdLacZ control group and also displayed recovery of both arterial driving pressure and microvascular haemoglobin oxygenation in contrary to the AdLacZ control.

### 3.4 Ischemic tissue damage is aggravated in the elderly, hyperlipidemic LDLR^−/−^ApoB^100/100^ mice with AdVEGF gene transfer

**FIGURE 4:**
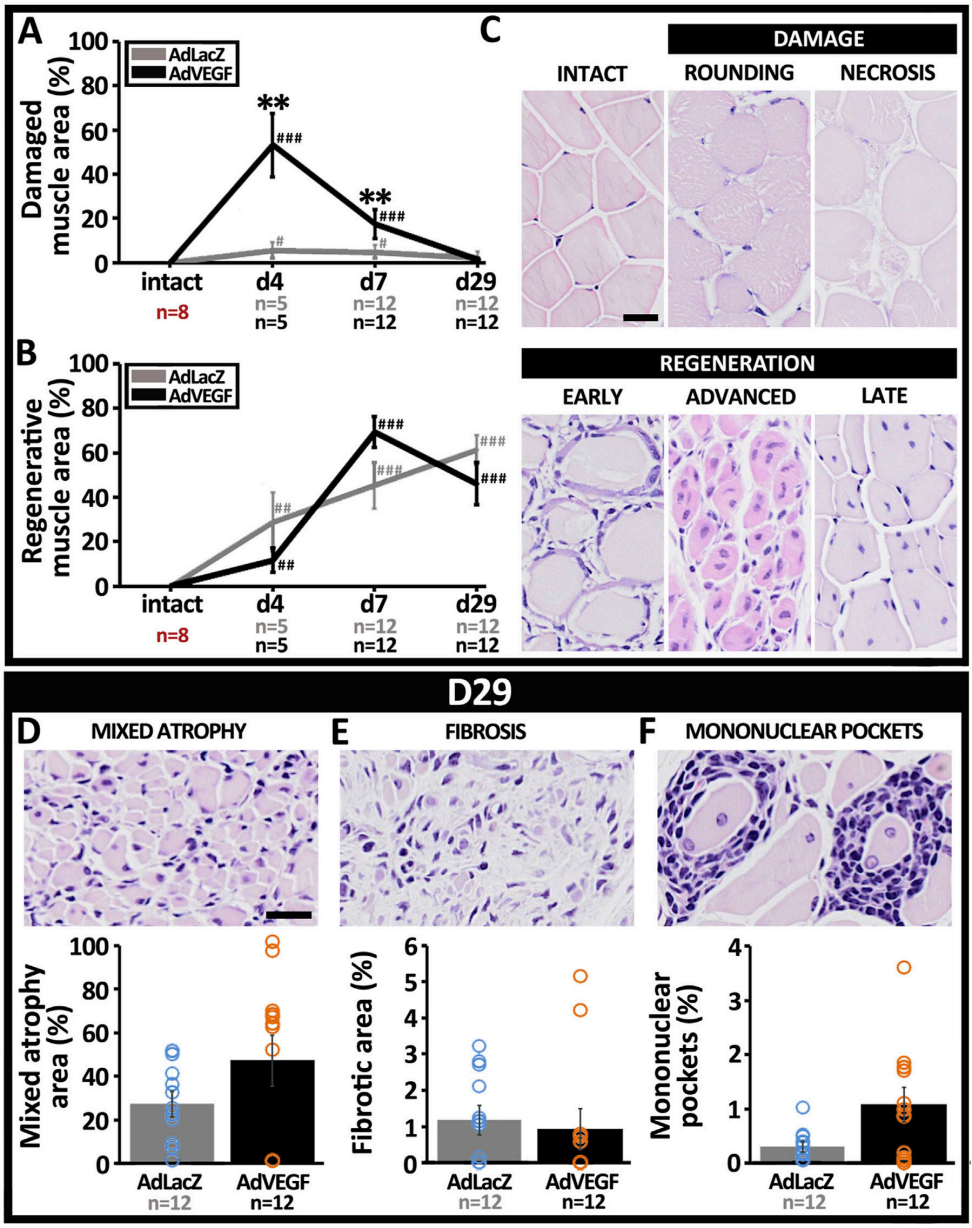
Ischemic muscle damage is aggravated by AdVEGF under hyperlipidemia. HE-based quantification of myofiber pathology revealed a significant increase in **(A)** ischemic damage after AdVEGF gene transfer at days 4–7 but no difference in **(B)** muscle regeneration between groups. **(C)** Representative HE stained images displaying morphological appearance of intact calf muscle and different types of ischemic myofiber damage and satellite cell-mediated muscle regeneration included in the analyses. Representative HE-images and quantification of areas with **(D)** mixed myofiber atrophy and **(E)** fibrosis, suggestive of chronic muscle damage. **(F)** Representative HE-image and quantification of mononuclear pockets inside the muscle, indicating possible immune response towards the adenoviral vector. Scale bars: 20 µm. Statistical significance is indicated by ^#^p < 0.05, ^**^, ^##^p < 0.01, ^###^p < 0.001. Comparison to AdLacZ is indicated with ^*^ and comparison to intact muscle with ^#^.

Based on visual examination, AdVEGF transduced hindlimbs were more often stiff and red than limbs in the AdLacZ group on days 4–7 (Table 1, P < 0.01). Also, macroscopic signs of severe ischemia, such as gangrene or paw autoamputation, were only seen in the AdVEGF group. Quantitation of HE-based muscle pathology (Figures 4A, B) displayed that on days 4–7 AdVEGF transduced ischemic calf muscles were significantly more damaged than AdLacZ muscles (Figure 4A, P < 0.01). The damage was predominantly presented as necrosis and scattered rounding of myofibers (Figure 4C). Regardless of differences in damage, areas of regeneration were similar in the groups during the 29-day follow-up (Figure 4B). In both groups, % of the muscle area under regeneration significantly increased over time, leaving around half of the calf muscle under regeneration on day 29. Beyond the common features of satellite cell-mediated muscle regeneration (Figure 4C), areas of mixed myofiber atrophy (Figure 4D) and fibrosis (Figure 4E) were found in muscle samples of both groups on day 29, indicative of chronic muscle damage. Additionally, pockets of mononuclear cell infiltrates (Figure 4F) were commonly found in muscle samples of both groups, possibly suggesting adaptive immune responses towards cells transduced by the adenoviral vector. Beyond possible damage caused by immune response towards the adenovirus on day 29, the angiogenic effects of the AdVEGF seemed to cause significant aggravation of ischemic damage during days 4–7.

**TABLE 1:** Prevalence of macroscopical ischemic symptoms in elderly, hyperlipidemic LDLR^−/−^ApoB^100/100^ mice after acute hindlimb ischemia and gene transfers. Limb stiffness/redness, gangrene, or paw autoamputation at different time points reported as number of animals/total animal number in each time point. AdVEGF transduced hindlimbs displayed more stiffness/redness than limbs in the AdLacZ group on days 4–7. Statistical significance is indicated by^**^p < 0.01,^***^p < 0.001.

	STIFFNESS/REDNESS				GANGRENE				PAW AUTOAMPUTATION			
	d1	d4	d7	d29	d1	d4	d7	d29	d1	d4	d7	d29
AdLacZ	0/16	12/20	5/17	1/9	0/16	0/20	0/17	0/9	0/16	0/20	0/17	0/9
AdVEGF	0/15	19/19	16/16	3/9	0/15	3/19	2/16	0/9	0/15	0/19	0/16	2/9
p-value	NS	**	***	NS	NS	NS	NS	NS	NS	NS	NS	NS

## 4 Discussion

In the present study, AdVEGF gene transfer was used as a gain-of-function approach in an attempt to rescue missing post-ischemic capillary enlargement and to improve muscle recovery in aged, hyperlipidemic LDLR^−/−^ApoB^100/100^ mice undergoing acute hindlimb ischemia. AdVEGF gene therapy has been generally considered beneficial for the treatment of skeletal muscle ischemia owing to its well-established biological properties to induce angiogenesis (Ylä-Herttuala et al., 2017). One of the biggest worries previously discussed in the context of clinical angiogenic trials has been the reduced potential of aged and diseased patients to respond to angiogenic stimuli (Gupta et al., 2009; Iyer and Annex, 2017). The results of this study suggest that the problem may not only be in the potential of the tissue to respond, but also the therapy itself. AdVEGF gene transfer here did induce capillary enlargement in the aged, hyperlipidemic LDLR^−/−^ApoB^100/100^ mice. Importantly, AdVEGF gene transfer even replicated the degree of capillary enlargement that was previously reported as the natural recovery response in ischemic skeletal muscles of aged, but healthy C57Bl/6J mice (Wirth et al., 2023). Furthermore in this study, AdVEGF-induced capillary enlargement in the LDLR^−/−^ApoB^100/100^ mice also assisted the normalization of arterial driving pressure similar to what was previously observed in the C57Bl/6J mice (Wirth et al., 2023). However, the AdVEGF gene transfer still did not improve post-ischemic muscle recovery.

AdVEGF-induced capillary enlargement here differed from the natural capillary response that was previously described in the C57Bl/6J mice (Wirth et al., 2023). Whereas the natural post-ischemic capillary enlargement in the C57Bl/6J was quick appearing already on day 1 (Wirth et al., 2023), the most prominent capillary enlargement in the AdVEGF group in this study was seen only on day 7. In addition to timing, also the type of capillary enlargement after AdVEGF gene transfer differed considerably from that previously described (Wirth et al., 2023). Enlarged, thick-walled capillaries after AdVEGF overexpression presented a striking contrast to transiently dilated, thin-walled capillaries observed previously in the C57Bl/6J (Wirth et al., 2023). The adenoviral-mediated chain of events, starting from viral entry into target cells and subsequent transgene expression to orchestration of angiogenesis, is much more complicated (Carmeliet and Jain, 2011; Rissanen and Ylä-Herttuala, 2007) and time-consuming than flow-mediated vasodilation that was previously suggested to mediate capillary dilation in response to acute ischemia in the C57Bl/6J (Wirth et al., 2023). This difference in timing may be critical not only considering the process of muscle recovery, but also the side-effects of angiogenesis (Korpisalo et al., 2011).

Angiogenic vascular growth is inclined to produce tissue edema as it requires the loosening of endothelial cell connections and increases vascular permeability (Weis and Cheresh, 2005; Bates, 2010). The adenoviral delivery of VEGF effectively asserts angiogenic stimulus in the target tissue over a time-period of at least several days leaving the vascular bed fully permeabilized (Korpisalo et al., 2011). AdVEGF gene transfer in the post-ischemic skeletal muscle here resulted in excessive edema formation on days 4–7. An increase in interstitial pressure caused by massive, persistent edema can compress tissue circulation (Weis and Cheresh, 2005) and likely explains the delayed recovery of microvascular blood flow in the AdVEGF transduced muscles. The lengthened duration of reduced tissue blood flow, again, logically explains the aggravation of ischemic tissue damage in the AdVEGF mice on days 4–7. Moreover, the adenoviral gene transfer itself seemed to cause mononuclear infiltrates in the transduced muscles. The control group here also displayed a significant increase in capillary size and tissue edema on day 7 as compared to its corresponding day 0 intact which were not detected in previous studies by Wirth et al. (Wirth et al., 2023). These changes could have reflected immune responses against the adenoviral vectors (Nayak and Herzog, 2010) which could have further complicated tissue recovery when compared to previous animals without gene transfers (Wirth et al., 2023).

Beyond edema, the long-lasting capillary enlargement induced by the AdVEGF could have also affected muscle damage. Damage related to capillary enlargement was recently reported in skeletal muscles of patients with chronic limb-threatening ischemia (CLTI) where myofiber atrophy was suggested to result from impaired oxygen diffusion caused by capillary enlargement (Tarvainen et al., 2023). In this study, the 29-day lasting enlargement in the AdVEGF animals could, therefore, have predisposed the muscles to chronic hypoxia. The adenoviral-mediated angiogenic changes have been commonly described to disappear after 2 weeks in healthy muscle (Rissanen et al., 2005; Korpisalo et al., 2011; Korpisalo et al., 2008a; Kholová et al., 2007). Adenoviral overexpression of VEGF alone is thus unlikely to explain the persistent capillary enlargement. Instead, e.g., growth stimuli released from the ischemic muscle of the hyperlipidemic mice, could have promoted persistent enlargement after the AdVEGF gene transfer, similar to CLTI (Tarvainen et al., 2023). Persistent capillary enlargement in the presence of low-pressure collateral mediated blood flow was also suggested in CLTI to cause microvascular shunting that could steal blood flow from normal capillaries (Tarvainen et al., 2023). In this study, during post-ischemic opening of collateral arteries, a gradually growing arterial driving pressure might be strong enough to fill the enlarged calf muscle capillaries but then likely not sufficient to propel blood further to the very distal parts of the swollen limbs. This could explain the two incidental autoamputations in the distal parts of the hindlimbs observed in this study after AdVEGF gene transfer at the day 29 time point.

The persistent capillary enlargement may also have interfered with muscle regeneration. Normalization of capillary size by intussusception and a subsequent increase in capillary density were previously identified to be essential for improving adequate tissue oxygenation during satellite cell-mediated muscle regeneration (Wirth et al., 2023). It has also been shown that increased blood flow initiates intussusceptive splitting of enlarged capillaries (Egginton et al., 2001; Styp-Rekowska et al., 2011). In this study, the delayed blood flow recovery after AdVEGF gene transfer could, therefore, have also contributed to the lack of intussusceptive splitting of enlarged capillaries. Consequently, the unchanged capillary density likely compromised tissue oxygenation and regeneration after AdVEGF gene transfer resulting in chronic damage similar to what has been recently described also in skeletal muscles of patients with CLTI (Tarvainen et al., 2023).

To conclude, hyperlipidemia or old age did not seem to impair AdVEGF-induced capillary enlargement. Importantly, the data from these experiments demonstrate also why the stimulation of persistent capillary enlargement may not serve to help skeletal muscle recovery from acute ischemic damage but rather leads to chronic damage under hyperlipidemia. Capillary enlargement has side-effects that can severely interfere with muscle blood flow, viability and even muscle oxygenation. Recapitulating the dynamic nature of capillary reactivity seems, therefore, essential for successful post-ischemic skeletal muscle recovery.

## Data Availability

The raw data supporting the conclusions of this article will be made available by the authors, without undue reservation.
